# An optimized optical-flow-based method for quantitative tracking of ultrasound-guided right diaphragm deformation

**DOI:** 10.1186/s12880-023-01066-7

**Published:** 2023-08-17

**Authors:** Qi Zhang, Dawei Yang, Yu Zhu, Yatong Liu, Xiong Ye

**Affiliations:** 1 School of Information Science and Engineering, East China University of Science and Technology, Shanghai, 200237 PR China; 2 Department of Pulmonary and Critical Care Medicine, Zhongshan Hospital, Fudan University, Shanghai, 200032 PR China; 3 Shanghai Engineering Research Center of Internet of Things for Respiratory Medicine, Shanghai, 200237 PR China; 4 School of Clinical Medicine, Shanghai University of Medicine & Health Sciences, Shanghai, 201318 PR China

**Keywords:** Diaphragm, Optical flow, Ultrasound, Strain, Deformation

## Abstract

**Objectives:**

To develop a quantitative analysis method for right diaphragm deformation. This method is based on optical flow and applied to diaphragm ultrasound imaging.

**Methods:**

This study enrolls six healthy subjects and eight patients under mechanical ventilation. Dynamic images with 3–5 breathing cycles were acquired from three directions of right diaphragm by a portable ultrasound system. Filtering and density clustering algorithms are used for denoising Digital Imaging and Communications in Medicine (DICOM) data. An optical flow based method is applied to track movements of the right diaphragm. An improved drift correction algorithm is used to optimize the results. The method can automatically analyze the respiratory cycle, inter-frame/cumulative vertical and horizontal displacements, and strain of the input right diaphragm ultrasound image.

**Results:**

The optical-flow-based diaphragm ultrasound image motion tracking algorithm can accurately track the right diaphragm during respiratory motion. There are significant differences in horizontal and vertical displacements in each section (p-values < 0.05 for all). Significant differences are found between healthy subjects and mechanical ventilation patients for both horizontal and vertical displacements in Section III (p-values < 0.05 for both). There is no significant difference in global strain in each section between healthy subjects and mechanical ventilation patients (p-values > 0.05 for all).

**Conclusions:**

The developed method can quantitatively evaluate the inter-frame/cumulative displacement of the diaphragm in both horizontal and vertical directions, as well as the global strain in three different imaging planes. The above indicators can be used to evaluate diaphragmatic dynamics.

**Supplementary Information:**

The online version contains supplementary material available at 10.1186/s12880-023-01066-7.

## Introduction

The diaphragm is composed of the top central tendon and muscle fibers radiating from the rib cage and diaphragm feet, and it’s a vault-like structure. And it is the largest skeletal muscle in the body. Its main function is to separate the thoracoabdominal cavity and maintain its pressure gradient. By changing the position of the diaphragm, it also performs normal respiratory movements. Diaphragmatic movements account for 60–75% of the work done by respiration [1], and during calm breathing, the top of the diaphragm drops 1.7 ± 0.2 cm, which corresponds to an increase in tidal volume of 758 ± 161 ml [2]. In a way, the normal contraction of the diaphragm is more important than the beating of the heart, because the heart’s contraction only delivers oxygen, while obtaining sufficient oxygen from the outside world must depend on the normal movement of the diaphragm. Without normal contraction of the diaphragm, the basic blood oxygen supply cannot be maintained. A variety of clinical factors, such as slow-onset lung disease, mechanical ventilation, cardiac-abdominal surgery, and phrenic nerve injury, can cause diaphragm dysfunction [3]. Once the function of the diaphragm becomes abnormal, it can lead to dyspnea, respiratory failure, and even death in severe cases [4].

Currently, the evaluation of diaphragm function is not as widely performed in clinical practice as to myocardial function. The assessments of diaphragm function available are mainly traditional dynamic evaluation methods such as transdiaphragm pressure, Chest X-ray, computed tomography, magnetic resonance imaging [5]. However, these methods have defects such as causing trauma and radiation exposure, and thus cannot be performed at the bedside. Therefore, it is necessary to find a non-invasive, economical, and convenient method for evaluating diaphragm function.

Ultrasound imaging (US) has advantages over X-ray fluoroscopy in terms of reducing radiation exposure to the patient and operator. Moreover, soft tissue structures such as nerves, muscles, tendons, and blood vessels can be visualized in real-time with US [6]. Thus, it is necessary to develop US-based methods for the evaluation of diaphragm function. However, current methods can only perform low-resolution video processing to achieve real-time video processing [7]. Since there is a trade-off between tissue inhomogeneity and precision resolution, large estimation bias and variance of ultrasound images are caused [8].

Initially, researchers evaluated the diaphragm function by calculating the amplitude of diaphragm movement with M-mode ultrasound [9, 10]. However, M-mode ultrasound can only conduct one-dimensional measurements and cannot continuously track the diaphragm movement [11]. Thus, B-mode ultrasound has also been used to measure diaphragm thickness as a measure of normal diaphragm motion [12, 13]. However, B-mode ultrasound only provides information on the structure of the diaphragm and does not reflect the mechanical properties of the diaphragm (i.e., elasticity information). Oppersma et al. [14] performed diaphragmatic strain analysis using existing ultrasound speckle-tracking software. The results showed that diaphragmatic strain was highly correlated with both trans-diaphragmatic pressure and diaphragmatic electrical activity (${r}^{2}=0.72$ and ${r}^{2}=0.60$). However, the software adopted in this method is specifically designed for myocardial strain analysis. Specifically, the adopted analysis trigger mode (with the appearance of ECG R wave as the trigger point) and duration (about 0.02s) are designed according to the myocardial contraction characteristics rather than the physiological characteristics of diaphragm respiration. With a respiratory rate of 12–20 times per minute and no pause in between, the diaphragm in the inspiratory phase contracts within 1.5–2.5 s, which is much longer than the analysis time of myocardial strain. In addition, this myocardial tracking algorithm is not open source, so it cannot be modified to accommodate diaphragm characteristics. For all of the issues mentioned above, it is not appropriate to use myocardial speckle-tracking software to study diaphragm movement patterns [15].

In order to optimize the performance of diaphragmatic strain imaging, this study proposes an optical flow (OF) based tracking method for the evaluation of diaphragmatic function using ultrasonic imaging. The proposed method is designed according to the characteristics of diaphragm movement while paying attention to the differences between the results obtained from different imaging views.

Our contribution can be summarized as follows:

- We propose a novel optimized optical-flow-based motion tracking algorithm for ultrasound images of right diaphragm deformation. The curve smoothing and drift correction methods are designed to improve the optical flow for diaphragm motion tracking. The algorithm can automatically transform qualitative analysis of diaphragm motion into quantitative analysis significantly.
- To obtain high-quality tracking points, we use filtering and the Density-Based Spatial Clustering of Applications with Noise (DBSCAN) algorithm to reduce the influence of ultrasound noise. It can effectively improve the performance of diaphragm motion tracking.
- We find significant differences between healthy subjects and mechanical ventilation patients by the quantitative results of diaphragm motion tracking. The method we proposed enables real-time processing of data, which has practical application value and can effectively assist physicians in clinical diagnosis of diaphragm disfunction.

## Materials and methods

### Data sources and definition of diaphragm displacement and strain

**Fig. 1:**
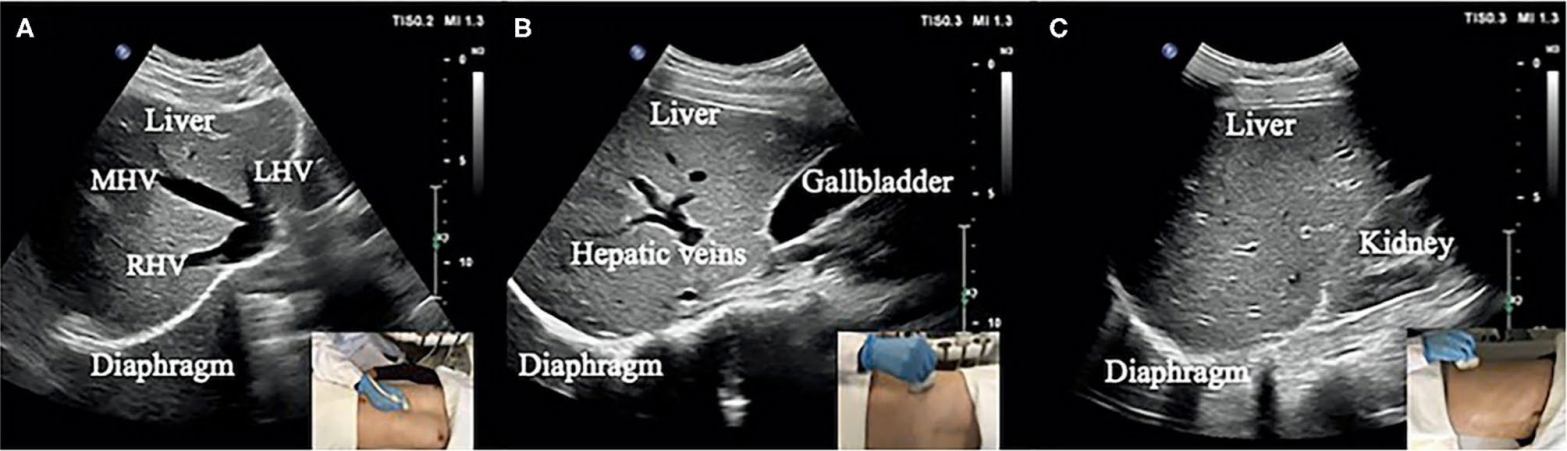
Three sections of ultrasound images. (**A**) Section I: Oblique section of the lower right costal arch through the second hepatic portal with the left hepatic vein (LHV), middle hepatic vein (MHV), and right hepatic vein (RHV) as anatomical markers. (**B**) Section II: Oblique section of the right intercostal passage through the first hepatic portal with the inferior vena cava, hepatic vein, and gallbladder as anatomical markers. (**C**) Section III: Sagittal section of the liver and right kidney with the right kidney and hepatorenal space as anatomical markers [16]

We use the same data as our previous work [16]. The movement of the diaphragm in three sections (Fig. 1) was assessed by a LOGIQ V2 ultrasound machine (General Electric Healthcare, Horton, Norway) with a 3–5-MHz convex-array probe.

Section I: Oblique section of the right costal arch through the second hepatic portal (Fig. 1A).

Section II: Oblique section of the right intercostal passage through the first hepatic portal (Fig. 1B).

Section III: Sagittal section of the liver and right kidney (Fig. 1C).

Two diaphragm deformation indicators are defined as follows:

#### Diaphragm displacement

**Fig. 2:**
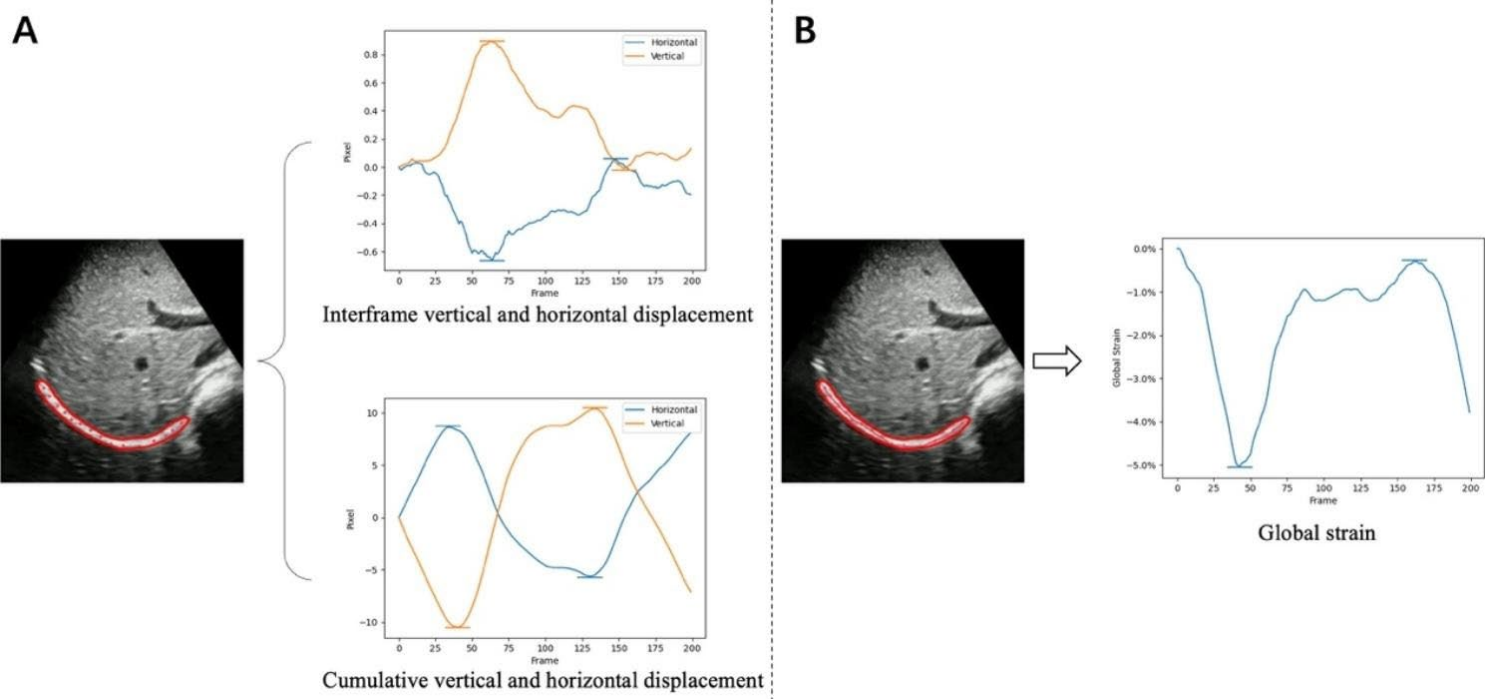
Definition of displacement and global strain. (**A**) Inter-frame vertical and horizontal displacement curves (top), cumulative vertical and horizontal displacement curves (bottom), and a schematic diagram of peak-to-peak extraction of the right diaphragm during one respiratory cycle. (**B**) Global strain curve of the right diaphragm and a schematic diagram of peak-to-peak extraction. The red region in the ultrasound image is the diaphragm area

Fifteen sampling points are generated in the diaphragm region (the generation process will be described in Sect. 2.3). As depicted in Fig. 2(A), there are two types of evaluations for diaphragm displacement based on different time intervals: (1) interframe displacement, which displays the movements (number of pixels) between two adjacent frames and is illustrated in the top diagram of Fig. 2(A); (2) cumulative displacement, which shows the movements between the current frame and the first frame and is illustrated in the bottom diagram of Fig. 2(A). Meanwhile, each diagram depicts two movement directions, namely vertical and horizontal, and the disparity between the maximum and minimum points in each direction represents the peak-to-peak value of the right diaphragm over one respiratory cycle.

#### Global diaphragm strain

The fifteen points are connected sequentially. We calculate the Euclidean distance between each two points and then add all the distances as an approximation of the diaphragm length. The initial length of the first frame is marked as $L\left(0\right)$, and the length at moment $t$ is recorded as $L\left(t\right)$, as shown in Fig. 2(B). The global strain (GS) is defined as:

1 $$\begin{array}{c}GS\left(t\right)=\frac{L\left(t\right)-L\left(0\right)}{L\left(0\right)}*100\% \end{array}$$

### Selection of tracking points in the diaphragm region

**Fig. 3:**
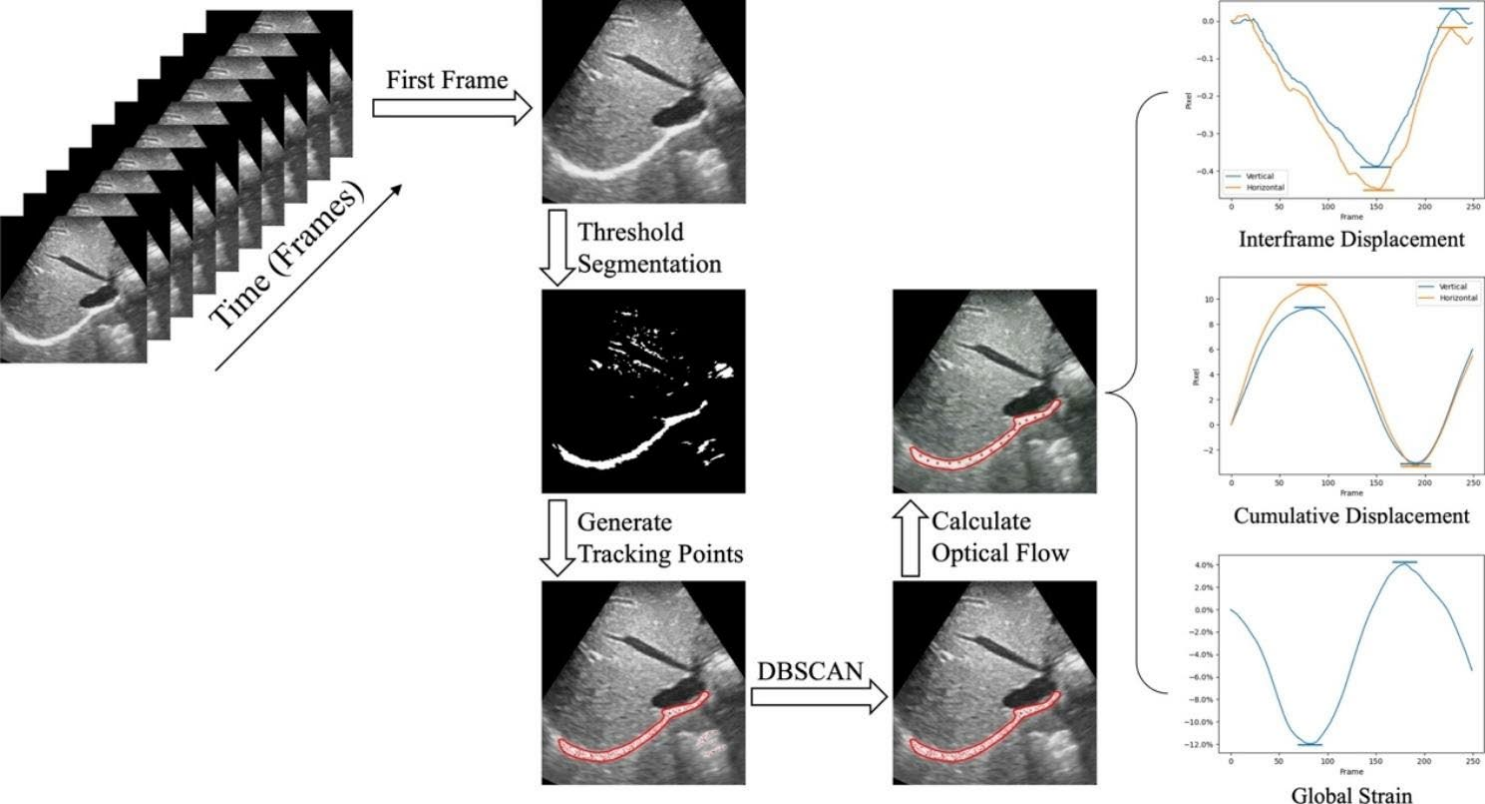
Flow chart of optical flow based tracking algorithm for diaphragm deformation evaluation. The red region in the ultrasound image is the diaphragm area

The flow chart of the whole proposed is shown in Fig. 3.

Due to the limitations of ultrasound imaging principles and recording equipment, ultrasound images typically exhibit low resolution, low tissue contrast, and high susceptibility to noise. Thus, filtering is required before analyzing the obtained ultrasound images. We use a simple 3 × 3 template for averaging filtering, and the averaging filtering formula is

2 $$\begin{array}{c}f\left(x,y\right)=\frac{1}{9}\sum _{j=y-1}^{y+1}\sum _{i=x-1}^{x+1}f\left(i,j\right) \end{array}$$

Where $f\left(x,y\right)$ is the pixel point in the image.

After filtering, the first frame of the ultrasound video is loaded, and a threshold $T$ is selected for the first frame for threshold segmentation:

3 $$\begin{array}{c}f\left(x,y\right)=\left\{\begin{array}{c}255, f\left(x,y\right)\ge T\\ 0, f\left(x,y\right) < T\end{array}\right.\end{array}$$

Where $f\left(x,y\right)$ is the pixel point in the image, 255 represents white, and 0 represents black. As the diaphragm region appears much brighter than other areas in ultrasound images, threshold segmentation can effectively differentiate it by assigning white to the diaphragm area and black to other irrelevant regions. The adaptive thresholding method is used for automatic segmentation, and the segmentation results can also be subsequently adjusted with manual assistance. After threshold segmentation, a maximum connected region is selected as the diaphragm. Then, the tracking point is located in the external rectangular box of the region.

**Fig. 4:**
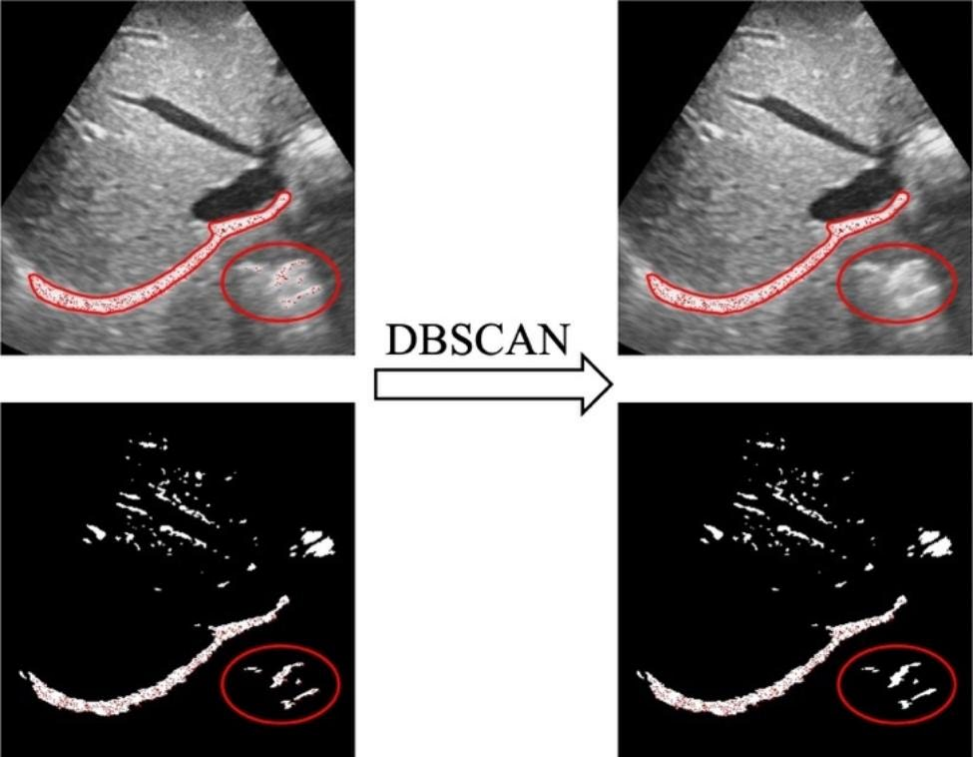
Remove irrelevant tracepoints using DBSCAN. The first row is the original image, and the second row is the corresponding thresholded segmented image

300 tracking points are randomly generated in the white area of the rectangular box. To remove tracking points from irrelevant regions, we utilize the Density-Based Spatial Clustering of Applications with Noise (DBSCAN) algorithm [17], as illustrated in Fig. 4.

### Calculation process of optical flow method

The two fundamental assumptions of the optical flow method are: (1) Luminance remains constant, meaning that the brightness of the target does not change as it moves between frames; (2) The continuity or motion in time is considered to be small motion, which implies that the changes in target position caused by time changes are not significant. In addition to the two basic assumptions above, the key assumption of the widely utilized Lucas-Kanade (LK) optical flow algorithm is spatial consistency. This means that adjacent points on the identical surface within the scene exhibit similar motion patterns and their corresponding projections on the image plane are in close proximity to one another [18]. According to the above three assumptions, we can obtain the optical flow vector as:

4 $$\begin{array}{c}\left[\genfrac{}{}{0pt}{}{u}{v}\right]={\left[\begin{array}{cc}\sum _{i=1}^{n}{I}_{xi}^{2}& \sum _{i=1}^{n}{I}_{xi}{I}_{yi}\\ \sum _{i=1}^{n}{I}_{xi}{I}_{yi}& \sum _{i=1}^{n}{I}_{yi}^{2}\end{array}\right]}^{-1}\left[\genfrac{}{}{0pt}{}{-\sum _{i=1}^{n}{I}_{xi}{I}_{ti}}{-\sum _{i=1}^{n}{I}_{yi}{I}_{ti}}\right] \end{array}$$

Where $I(x,y,t)$ is the brightness of the image at position $\left(x,y\right)$, ${I}_{x}$, and ${I}_{y}$ are the partial derivatives of $I$ to $x$ and $y$, respectively. $u$ and $v$ are the derivatives of the pixel points along the $x$ and $y$ directions, respectively, i.e., $\frac{\delta x}{\delta t}$ and $\frac{\delta y}{\delta t}$. $n$ is assumed to be constant for the brightness of the image within an $m\times m$ window of the specified size, i.e., $n={m}^{2}$.

For all retained key points, their positions in the next frame are calculated one by one. The offset between two frames can be decomposed into horizontal and vertical displacements. The frame displacement is then determined by calculating the average of all point displacements. The inter-frame and cumulative displacement curves of the diaphragm can be calculated sequentially until the end of the ultrasonic video. All the key points are divided into 15 groups equally, and the center point of each group is selected as a key point for GS calculation. The length obtained by sequentially connecting 15 central points is used as the GS indicator for the diaphragm, after which the strain curve of the diaphragm can be obtained.

### Curve smoothing and drift correction

Due to noise interference in the ultrasound image and inaccuracies in the algorithmic calculations, the resulting output curve may exhibit jitter. To obtain a smoother two-dimensional output curve, a convolutional smoothing technique is employed. Specifically, a one-dimensional convolutional kernel with a size of 5 is applied to calculate the curve point by point. After curve smoothing, a complete respiratory cycle can be obtained according to the peak and trough of the curve:

5 $$\begin{array}{c}T=2*\left|adj\left(pk-th\right)\right|\end{array}$$

Where $T$ represents the respiratory cycle, $adj(\cdot)$ represents the operation of taking the adjacent wave peaks and troughs, $pk$ represents the wave peaks, and $th$ represents the wave troughs.

Since the estimation error will be accumulated during the tracking process, the output displacement and GS curves will drift as the number of frames increases [19]. Therefore, a drift correction algorithm is used to compensate for displacement and GS. In this study, we improved the drift correction method based on Ye et al. [16]. As tracking progresses, the drift will inevitably increase. To mitigate this issue, the first respiratory cycle is used as the reference point. It is expected that the diaphragm will return to its starting position at the end of each complete cycle. We define the correction as the state at time $t$ minus the offset at the end of the first cycle multiplied by the current moment, as the following equation:

6 $$\begin{array}{c}{S}_{corr}\left(t\right)=S\left(t\right)-\frac{S\left(T\right)}{T}\times t\end{array}$$

**Fig. 5:**
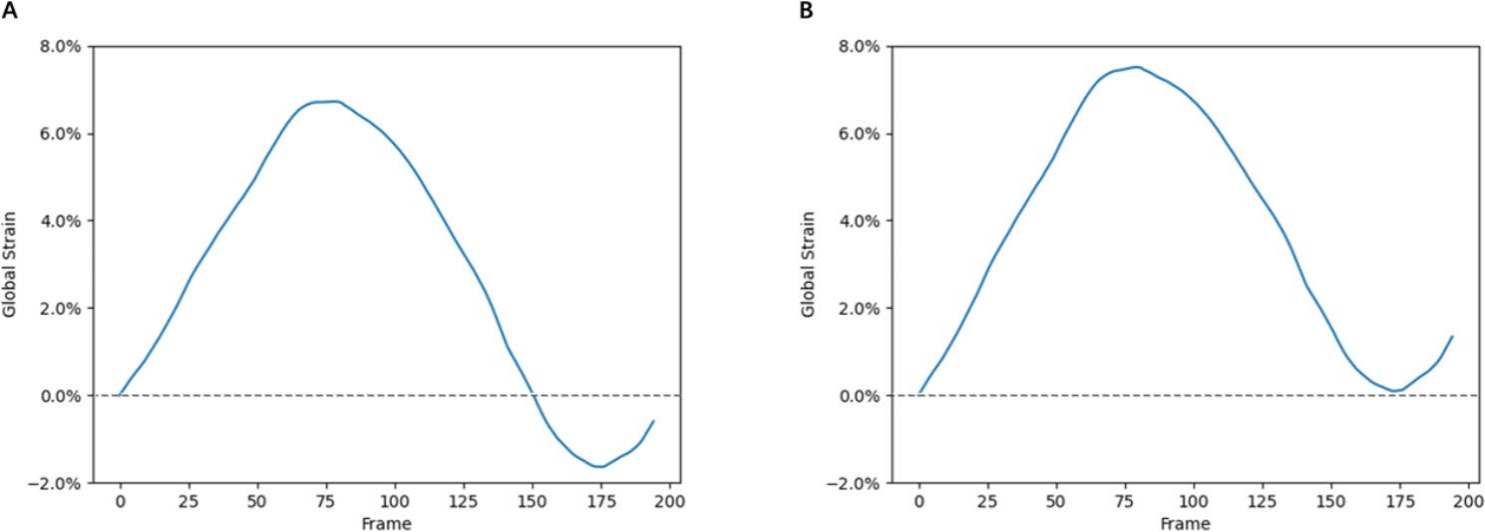
(**A**) GS curve before drift correction. (**B**) GS curve after drift correction

Where ${S}_{corr}\left(t\right)$ is the displacement after correction and $S\left(t\right)$ is the displacement before correction. When $t=nT (n=1, 2, 3, \dots )$, $S\left(T\right)$ should return to the original position (i.e., the position where $S\left(t\right)=0$). Figure 5 shows the results before and after drift correction. It can be seen that our improved method solves the drift problem effectively and can return to the initial position after the respiratory cycle.

Finally, the corrected inter-frame and cumulative displacement curves of the diaphragm and the GS curve of the diaphragm are output respectively.

### Data analysis

We used SPSS version 25 (SPSS Inc., 2016, Armonk, NY) for all analyses. All variables were tested for normality before data were presented as either the mean ± standard deviation (SD). Differences between the two groups were compared using unpaired t-tests. P-value < 0.05 was considered dominant.

## Results

### Basic information of subjects

Diaphragm ultrasound images were acquired from six healthy subjects during quiet spontaneous breathing and eight patients under mechanical ventilation, all of whom were males. The basic information of all subjects is shown in Table 1. The study subjects are consistent with our published paper [16].

**Table 1:** Basic information of 14 subjects (Mean ± SD) [16]

Variable	Healthy subjects (N = 6)	MV patents (N = 8)	P
Age (years)	56 ± 8.2	51 ± 3.6	0.061
BMI (kg/m2)	21 ± 5.3	23 ± 2.8	0.084
Tidal volume (ml)	520 ± 17.3	450 ± 38.2	0.102
Ventilation mode	-	SIMV	-

Note: BMI, body mass index; MV, mechanical ventilation; SIMV, synchronized intermittent mandatory ventilation

### Continual tracking of diaphragm movement

**Fig. 6:**
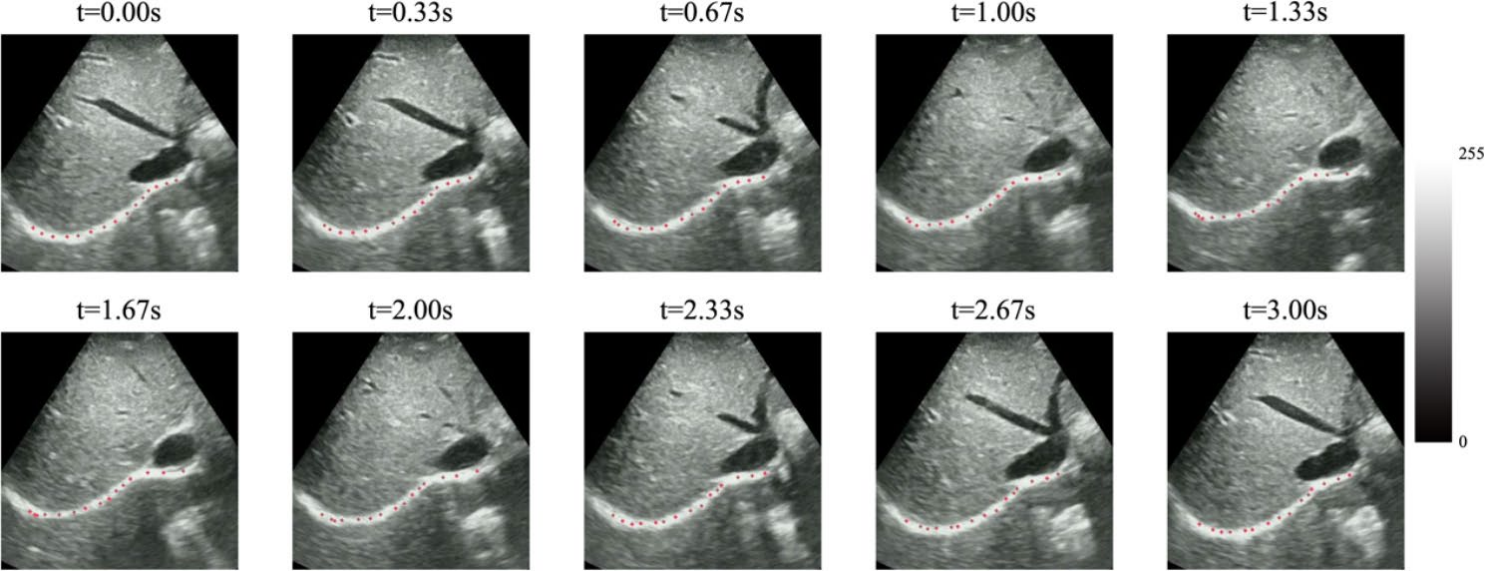
The results of the optical-flow-based diaphragm deformation motion tracking algorithm during one respiratory cycle

Figure 6 shows the results of the optical-flow-based diaphragm deformation motion tracking algorithm that continuously tracks the diaphragm motion during one respiratory cycle. It can be seen that the diaphragm at the moment $t=1.33s$ is shorter than that at $t=0s$, which indicates that the diaphragm is in the inspiratory contraction phase during this cycle. From the moment $t=1.33s$ to $t=3s$, the diaphragm is slowly stretched and eventually returns to its initial position.

### Displacement of the diaphragm

**Fig. 7:**
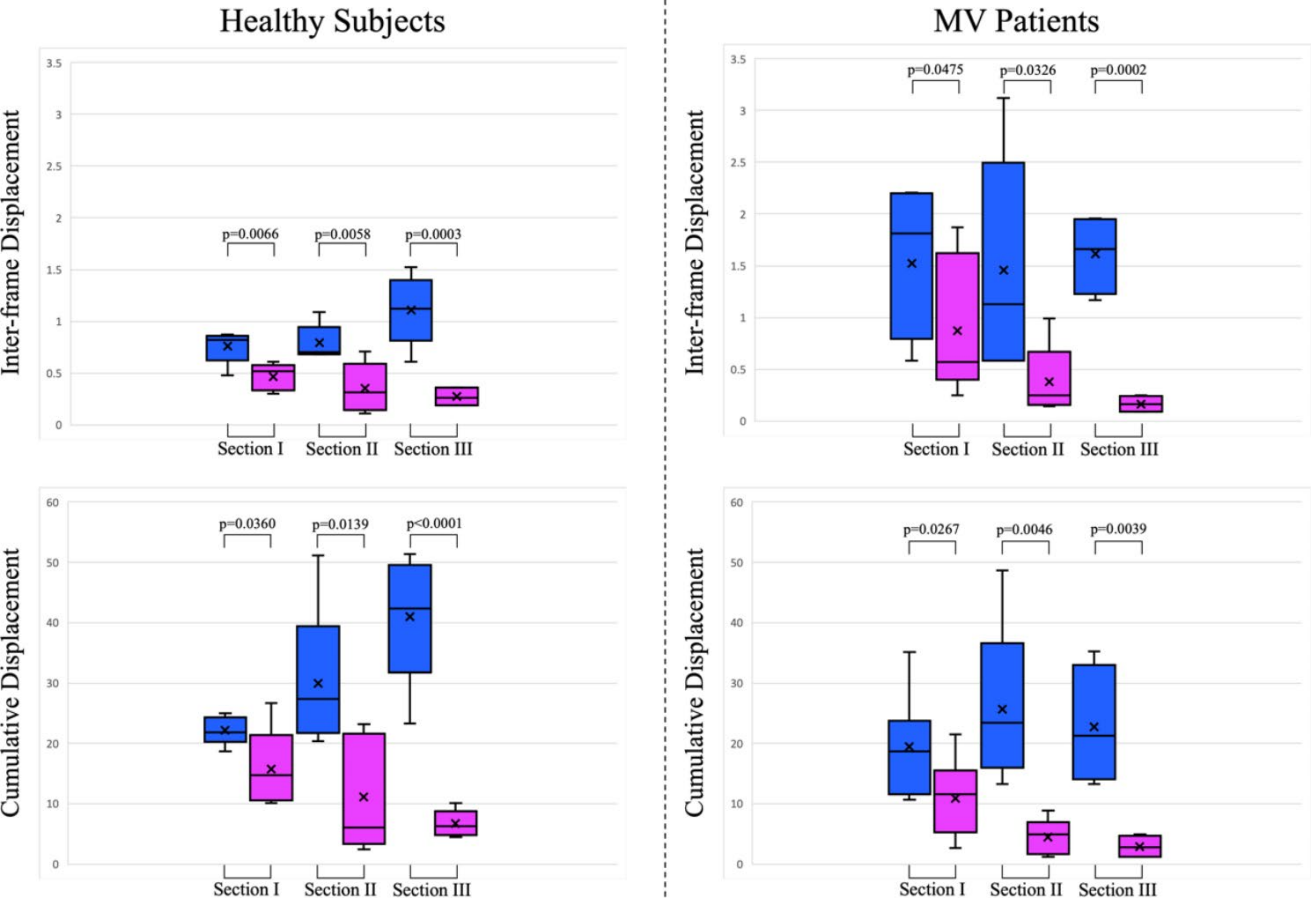
Inter-frame and cumulative horizontal (blue) and vertical displacement (purple) box-plot of the right diaphragm for the same ultrasound imaging section in healthy subjects (left) and mechanical ventilation patients (right)

Figure 7 depicts the horizontal and vertical interframe and cumulative displacements of the same section of the right diaphragm in healthy subjects and mechanical ventilation patients, respectively. Blue and purple represent the horizontal and vertical displacements of sections I, II, and III, respectively. The p-values of the differences between the horizontal and vertical displacements of sections I, II, and III of healthy subjects are 0.0066, 0.0058, and 0.0003, respectively, and the p-values of the differences between the cumulative horizontal and vertical displacements are 0.0360, 0.0139 and < 0.0001, respectively. The p-values of the difference between the horizontal displacement and vertical displacement of sections I, II, and III of mechanical ventilation patients are 0.0475, 0.0326, and 0.0002, respectively, and the p-values of the difference between the cumulative horizontal displacement and vertical displacement are 0.0267, 0.0046 and 0.0039, respectively. All of these are significantly different.

**Fig. 8:**
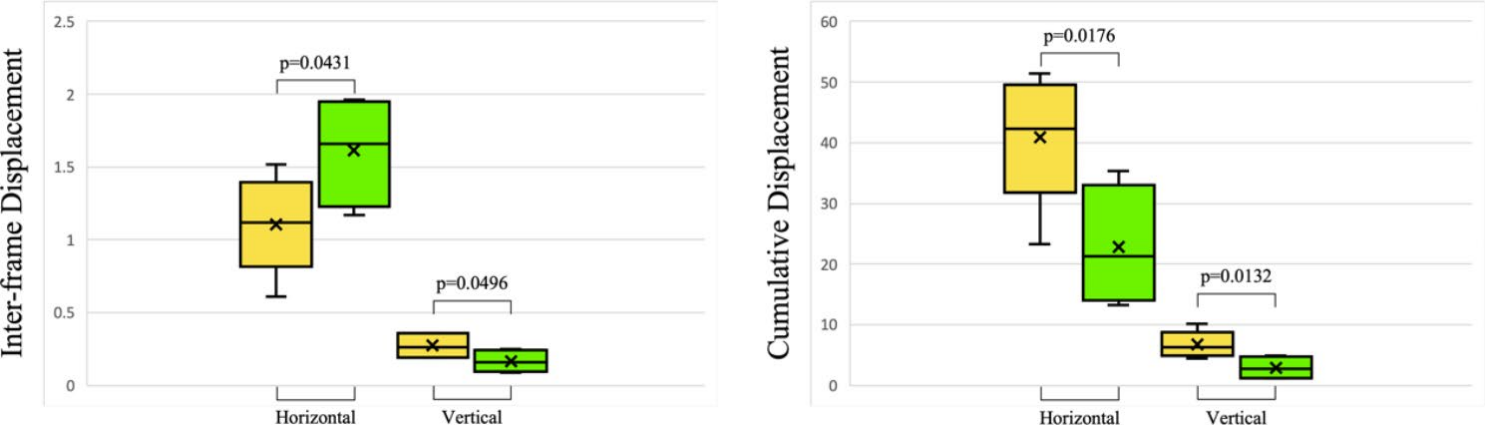
Inter-frame and cumulative horizontal and vertical displacement box-plot of diaphragm ultrasound imaging Section III in healthy subjects (yellow) and mechanical ventilation patients (green)

In addition, we find that for healthy subjects and mechanical ventilation patients, there are significant differences in the four dimensions of horizontal and vertical displacement, the cumulative horizontal and vertical displacement between Section III, as shown in Fig. 8. Yellow and green represent healthy subjects and mechanical ventilation patients, respectively. The p-values of the differences in inter-frame horizontal and vertical displacements between healthy subjects and mechanical ventilation patients in Section III are 0.0431 and 0.0496, respectively. The p-values of the differences in cumulative horizontal and vertical displacements are 0.0176 and 0.0132, respectively. All of these are significantly different. Therefore, in practice, Section III can be used to determine healthy subjects and mechanical ventilation patients.

### Global strain (GS) of the diaphragm

Table 2 shows the GS of the right diaphragm of healthy subjects and mechanical ventilation patients in three sections. And obviously, there is no significant difference in the global strain of each section between healthy subjects and mechanical ventilation patients (p > 0.05).

**Table 2:** GS of the subject’s right diaphragm

Section	Class	GS(%) (Mean ± SD)	95% CI	p
I	Health Subjects	9.68 ± 3.25	5.64–13.72	0.1160
	MV Patients	12.43 ± 4.22	8.53–16.33	
II	Health Subjects	12.10 ± 2.57	8.91–15.29	0.3802
	MV Patients	13.30 ± 6.94	2.26–24.34	
III	Health Subjects	13.68 ± 3.32	9.56–17.80	0.1094
	MV Patients	10.05 ± 4.32	3.18–16.92	

## Discussion

The diaphragm serves as the main respiratory muscle pump and is widely distributed between the abdominal and thoracic regions, including the crural, dorsocostal, midcostal, and ventrocostal regions, as well as the zone of apposition [20]. Functional disorders of the diaphragm can lead to respiratory distress in patients, and the diaphragm has important physiological functions. Therefore, it is necessary to evaluate and monitor the changes in its function in clinic [21, 22]. In ultrasound strain imaging, the displacement and deformation of tissue are estimated using pre- and post-compression imaging data [23].

In this study, we develope a new optical-flow-based diaphragm motion tracking algorithm and successfully analyzed the deformation characteristics of the diaphragm. As shown in Fig. 6, the entire process of active contraction of the right diaphragm during the inspiratory phase and passive relaxation during the expiratory phase of a respiratory cycle can be observed. During one respiratory cycle, our algorithm continuously tracks the vertical and horizontal movements of the right diaphragm as well as the GS. Our improved drift correction algorithm can better mitigate the drift in motion tracking. To our knowledge, this is the first study to analyze diaphragm deformation using a newly designed algorithm based on optical flow, which uses the knowledge of diaphragm structure, motion mechanism, and ultrasonic imaging principle. The calculation time of our method is related to the number of input video frames. It takes about 32 milliseconds to calculate the optical flow between two frames on average. The short calculation time makes it possible to translate this diaphragm motion tracking algorithm into clinical practice. In all three sections, we utilized the peak-to-peak values of inter-frame and cumulative displacements, as well as the GS of the right diaphragm, as indexes for analyzing diaphragm kinetics. The dynamic index of GS reflects the dynamic characteristics of the diaphragm during respiration in a graphical manner, demonstrating the tissue characteristics of the diaphragm during active or passive contraction during respiration.

There are some findings in this study. First, the inter-frame displacement of healthy subjects is lower than that of mechanical ventilation patients, but the cumulative displacement of healthy subjects is higher than that of mechanical ventilation patients. Secondly, in section III, it is found that there are significant differences (p < 0.05) in the horizontal and vertical displacement between healthy subjects and mechanical ventilation patients, both in inter-frame and cumulative time intervals. These differences can be utilized in clinical settings to differentiate between healthy subjects and mechanically ventilated patients. Another finding is that there is no significant difference in the GS between healthy subjects and mechanical ventilation patients in all three sections (p > 0.05). This is because the amplitude of diaphragm movement determines the size of the tidal volume, while Table 1 shows that there is no significant difference between the baseline tidal volume of healthy subjects and mechanical ventilation patients (p > 0.05). Furthermore, there is no significant difference between the results of our algorithm and those of Ye et al. [16] (p > 0.05), which shows the effectiveness of our algorithm.

The proposed method is superior to three aspects of existing diaphragm motion assessment methods. Firstly, our method has the benefit of automatically selecting the diaphragm region, while also boasting a faster calculation speed (specifically, optical flow calculation is quicker than interpolation calculation in our previous work [16]). Secondly, we improved the drift correction algorithm in our previous work [16] based on the characteristics of the optical flow tracking results. The most important point is that our proposed method can perform real-time processing of diaphragm ultrasound images input to the program, which is crucial in medical diagnosis.

There are some limitations to this study as well. Firstly, the number of data samples involved in algorithm verification is relatively small. Moreover, due to the inherent challenge of calculating optical flow, there is still potential for further optimization of the global strain of the diaphragm. Meanwhile, the tracking of the segmental motion of the diaphragm has not been studied. The overall strain calculation of the diaphragm muscle and tendon may be canceled out by opposite movements. Therefore, future studies should consider subdividing the diaphragm region into muscle and tendon segments.

## Conclusions

In conclusion, we develope a novel tracking method of diaphragm deformation (displacement and strain) based on optical flow. The validity of this method is confirmed by analyzing diaphragmatic function and dynamics indexes. We examine the horizontal and vertical movements in inter-frame and cumulative time intervals, as well as the discrepancies in GS measurements of the right diaphragm across three sections. The results show that the horizontal displacement of the diaphragm is greater than the vertical displacement in each section. Healthy subjects and mechanical ventilation patients can be distinguished in Section III. The GS of the diaphragm is not sensitive to imaging sections. In subsequent studies, more clinical data need to be collected to further explore the value and significance of this new technology.

### Electronic supplementary material

Below is the link to the electronic supplementary material.

Supplementary Material 1

Supplementary Material 2

## Data Availability

The data that support the findings of this study are available from the corresponding author upon reasonable request.
