# Supplementary material for: An optimized optical-flow-based method for quantitative tracking of ultrasound-guided right diaphragm deformation
Source: BMC Med Imaging. 2023 Aug 17;23:108. doi: 10.1186/s12880-023-01066-7 (PMC10436632; doi:10.1186/s12880-023-01066-7)
Supplement: Supplementary file 2 — Supplementary Material 2 [file 12880_2023_1066_MOESM2_ESM.docx]

The uploaded video is the result of the algorithm we proposed to track ultrasound images of the diaphragm. The red points in the video are the key points that we automatically extracted using our algorithm. We can see that our proposed algorithm is able to continuously track the movement of the diaphragm as it continues.
